# Annotations

**Published:** 1895-02-16

**Authors:** 


### ANNOTATIONS.

### Infectious Catarrh.

THERE is something very interesting in the experience of the staff of the observatory on the summit of Ben Nevis in regard to their susceptibility to catarrh. One might have expected that exposure to such inclemencies of climate as must prevail in such a situation would have led to perennial "colds," instead of which it is stated that while in actual residence on the summit the staff have, for the eleven years which have elapsed since the observatory was established, been remarkably free from ailments of all sorts. While residing at the low level, however, as they do in turn, they seem peculiarly subject to a sort of influenzal catarrh. This commences about forty-eight hours after coming down and lasts for from ten days to three weeks. No protection is afforded against future attacks, and these are as apt to occur after one month's residence at the summit as after three months. It is interesting also to observe that if an individual joins the staff on the summit while suffering from a cold he almost surely infects them also with catarrh, which, however, seldom endures longer than twenty-four hours. From an epidemiological point of view this observation is of considerable interest. The catarrh in question is evidently an infective disease of a zymotic nature, and the organisms on which it depends must be practically continuously present down below. Why, then, do those who dwell in this region of endemic catarrh not suffer from it more or less continuously? The suggestion clearly is that continued exposure to infection maintains an immunity, once produced, even where this immunity is so short-lived as it evidently is in the case we are considering. If this is so, it would but tend to confirm what has long been suspected in regard to those infectious diseases which especially attack new-comers to their endemic homes, such as yellow fever, cholera, and certain forms of typhoid prevalent in India, viz., that prolonged exposure to the virus leads to a certain degree of insusceptibility to it. Facts of this nature are of much importance in elucidating the causes of the dying down of epidemic outbreaks of disease.

### Mr. Balfour's "Belief."

IN the process of human development it has been observed that "the savage argues with his club; the man of science with his pen." In quite recent times we have enlarged the definition of "the man of science." By way of recreation after his Parliamentary labours, also perhaps by way of the better fitting himself for those labours, Mr. Balfour has carried through a scientific investigation of the "Foundations of Belief." Why does a man believe? In ultimate analysis he believes because he cannot help it. We used to be told that the only solid foundations of belief were those we could see and handle. All else was guesswork. Mr. Balfour now tells us that most of the conclusions we arrive at about the things we see and handle are guesswork too. The eyes and the fingers can tell us something about things which can be seen and handled, but they cannot tell us all about those things. Moreover—and this is a crucial point—we have no means of knowing how far what they do tell us is "the truth, the whole truth, and nothing but the truth," as the lawyers have it. The eye bears what witness it can, but it is only a relative, not an absolute witness. So with the finger Mr. Balfour contends that as it is with the things of the senses so it is with the things of the mind. The senses make the best efforts they can to deal with the things which come within their cognisance, and so does the mind. But neither the one nor the other knows exactly, or knows entirely. It is even so. The science of cerebral physiology confirms it all. Therefore the man who believes in the things which impress themselves upon his convictions as worthy of belief, stands on the same foundation as the man, hitherto called "the man of science," who believes what he sees and feels, and what he deduces from his seeing and feeling. It is all there. The religious man, who is strictly honest, has precisely the same kind of foundations for his sincere beliefs as the man of science who is strictly honest has for his: no more and no less.

### Sewage Farming in India.

SOME points brought into prominence by Mr. J. Nield Cook, in his recent paper read before the members of the Indian Medical Congress, appear to us to be of first-rate importance. They incidentally prove two things which bear upon the disposal of sewage in this country. In the first place, they show under what conditions sewage-farming is likely to fail; and in the second, they tell us under what conditions—in India—such farming has been highly profitable in itself, and of great advantage to the towns from which the sewage has been supplied. Sewage-farming is likely to fail on heavy soils, and in flat or marshy districts. Such soils and situations both retain too much of the sewage, and for too long a period. On the other hand, light porous soils, on gentle declivities which allow of the free percolation downwards of the liquid portions of the sewage after filtration through the soil, are very favourable conditions. In the neighbourhood of Madras sewage farming has been carried on for twenty-five years, with the grand result that an almost unlimited amount of sewage has been disposed of, and the produce of the farms has found ready markets and realised handsome profits. In situations that are really favourable for sewage farming extraordinary crops and profits are recorded. For example, as much as sixty-nine tons per acre of grass has been known to be produced in a single year; not of course in a single crop. As a matter of fact, in some parts of India seven or eight crops in the year are sometimes grown on well-managed sewage farms, and crops, be it remembered, of thoroughly sweet and wholesome produce. Young grass, it appears, can take enormous quantities of sewage. On the other hand, when the grass is just becoming ready for cutting, it is better not to give it any sewage at all. With regard to the healthiness of the surrounding atmosphere and of persons employed on sewage farms, complaints are practically unknown. Given the three conditions of a porous soil, a gentle declivity, and a good daily average of sunshine, it would seem that sewage farming ought to do well in any country where grass is worth the growing.

